# Towards the Internet of Flying Robots: A Survey

**DOI:** 10.3390/s18114038

**Published:** 2018-11-19

**Authors:** Hailong Huang, Andrey V. Savkin

**Affiliations:** School of Electrical Engineering and Telecommunications, University of New South Wales, Sydney 2052, Australia; hailong.huang@unsw.edu.au

**Keywords:** flying robots, target tracking, monitoring and surveillance, wireless sensor networks, flying robot navigation, collision avoidance, path planning, Internet of Things

## Abstract

The Internet of Flying Robots (IoFR) has received much attention in recent years thanks to the mobility and flexibility of flying robots. Although a lot of research has been done, there is a lack of a comprehensive survey on this topic. This paper analyzes several typical problems in designing IoFR for real applications, including wireless communication support, monitoring targets of interest, serving a wireless sensor network, and collaborating with ground robots. In particular, an overview of the existing publications on the coverage problem, connectivity of flying robots, energy capacity limitation, target searching, path planning, flying robot navigation with collision avoidance, etc., is presented. Beyond the discussion of these available approaches, some shortcomings of them are indicated and some promising future research directions are pointed out.

## 1. Introduction

**Figure 1:**
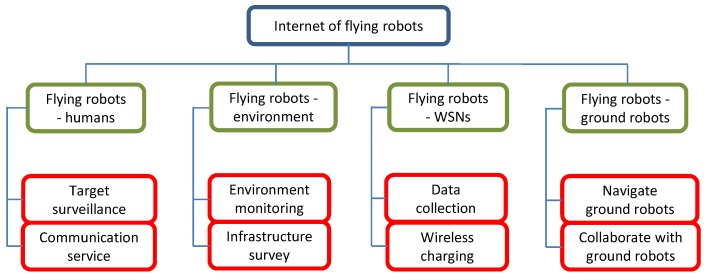
Applications of FRs.

The Internet of Flying Robots (IoFR) is a sub-concept of the Internet of Things (IoT) [1,2] and Internet of Robotic Things [3]. The flying robots (FRs) may refer to drones, Unmanned Aerial Vehicles (UAVs), or airships in different applications [4]. Thanks to the inherent attributes such as mobility and flexibility of FRs, IoFR has been widely used in recent years, including some conventional applications such as military, rescue operations, surveillance, and monitoring, and also some emerging applications such as goods delivery and telecommunications [5]. Some typical applications of IoFR are summarized in Figure 1. Firstly, FRs can service humans. For example, they can play the role of aerial base stations to provide communication service to cellular users, especially in some congested urban areas [6]. This is a promising solution to 5G or beyond-5G networks. Also, FRs can be used to track targets, such as humans, animals, and vehicles [7]. Secondly, FRs are used in agriculture [8], traffic monitoring [9], architecture inspection [10], environment monitoring [11], disaster management [12], etc. Furthermore, FRs can service wireless sensor networks (WSNs) [13]. Working as the flying mobile sinks, these robots can collect sensory data from distributed and/or sparsely deployed sensor nodes; and working as chargers, they can wirelessly recharge sensor nodes’ batteries, in order to prolong network lifetime. Moreover, they can navigate ground robots since they may have a better view of the environment, and they can also collaborate with ground robots to execute complex tasks. Beyond those listed in Figure 1, delivery service is another application attracting interest from both the research community [14] and business community. For example, a startup company has been focusing on applying FRs to deliver medicine in Rwanda [15].

Despite having many promising opportunities, several key issues must be addressed to facilitate the deployment of IoFR. The first issue is coverage. Generally, the system providers wish to provide a system which has a sufficient coverage of targets, either humans or field sites. The second issue is connectivity, specifically, forming a connected network enables fast information transmission between FRs and the ground control stations. The third issue is about energy. Since most commercial FRs currently available on the market are powered by batteries, working time is limited. Thus, how to ensure persistent service is another aspect of quality of service.

### 1.1. Motivation

Extensive research relevant to FRs has already been conducted and there are some survey papers. To clarify the position of this paper, the relevant survey papers are reviewed.

The survey [16] provides a summary of the commercial, open-source and research autopilot systems for the convenience of potential small FR users. The survey [17] pays attention to the flying ad-hoc networks, as a new family of networks. Instead of being restricted by infrastructure, FRs can construct ad-hoc networks to address many infrastructure-related issues. Reference [18] also considers communication issues in flying robotic networks. Beyond [17], issues of self-organization, disruption tolerance, SDN control, seamless handover, and energy efficiency have been discussed in [18]. Reference [19] overviews the FR-based communication system and outlines various protocols that can be used at different FR communication links and networking layers. Also, it discusses the approaches of using FRs to collect data from WSNs. Survey [20] overviews three specific applications of FRs: FR-aided ubiquitous coverage, i.e., FRs are deployed to assist the existing communication infrastructure; FR-aided relaying, where FRs are deployed to provide wireless connectivity between distant users or user groups without reliable direct communication links; and FR-aided data dissemination (collection), where FRs are sent out to disseminate (collect) data to (from) a set of distributed wireless nodes. Survey [12] reviews the usage of FRs for imagery collection in disaster research and management. This paper presents a review of publications describing data acquisition and assessment prior, during and after disaster events. The survey [8] focuses on the application of FRs in agriculture. Compared to the high cost of satellite images, low-altitude FRs can also take photos with high spatial and temporal resolution to identify variations in the field. Publication [9] considers the application of FRs on traffic monitoring and management. Another recent survey [21] reviews recent publications following the types of FR operations, such as area coverage, target searching, routing for a set of locations, etc.

From the overview of the existing survey papers on the topic of FRs, one can see that the main focus of [16] is on the control perspective, including the flight control, radio control system and autopilot control system in both hardware and software, which are some classic topics. The main focus of [17,18,19] lies in communications. Surveys [8,9,12,19,20] have discussed some applications of FRs. It is clear that compared to the applications listed in Figure 1, these papers only review a subset of potential applications. Survey [21] is based on the taxonomy of FR operations, which does not provide insights into similarities of those operations. Although FRs have various applications, some common issues exist across them and if these issues can be abstracted and analyzed comprehensively, system designers will benefit. Therefore, there is a necessity to review the available approaches from a new point of view: surveying the common issues across different applications, discussing approaches which are available to address them, analyzing their merits and shortcomings, and pointing out some promising solutions to improve them if possible.

### 1.2. Contribution

In this paper, the available approaches are categorized based on type of application. This is achieved by analyzing similarities among various applications. From the point of objects being served, there are two groups: collaborative objects and non-collaborative objects. Specifically, collaborative objects refer to those which can collaborate with FRs. For example, as shown in Figure 1, the applications corresponding to this kind of object is using FRs to navigate ground robots. Non-collaborative objects refer to those which only accept service passively, such as cellular users, sensor nodes, and the fields to be monitored. Then, potential applications include target surveillance, providing communication service, and charging sensor nodes.

The non-collaborative group consists of varying objects and the tasks of FRs are also different in varying applications. However, there are some common problems across these applications. The most important problem is coverage. In the application of support SBSs for wireless communications, FRs need to provide coverage to mobile users which guarantee high quality of service; in the application of target tracking, FRs need to observe all the targets continuously; and in the application of filed monitoring, FRs may need to scan the field frequently and repeatedly. Some other common issues include the requirement of connectivity of FRs and their energy limitation. With this consideration, this paper mainly discusses these issues for the non-collaborative group. For the collaborative group, this paper focuses on the collaboration with ground robots and WSNs. The typical approaches for localization of FRs, path planning, collision avoidance navigation, etc., are discussed.

Based on discussions, some comments on the existing approaches are further provided, their limitations are pointed out, and some promising future research directions are suggested.

### 1.3. Paper Organization

The rest of the paper is organized as follows. Section 2 reviews approaches related to applications involving the first type of objects, i.e., those passively accepting service. In particular, this section focuses on common issues including coverage, connectivity, and energy in these applications. Section 3 reviews approaches for the second type of objects, i.e., which can collaborate with FRs. It concentrates on two systems: the flying-ground robotic system, and the system consisting of FRs and a WSN. Section 4 presents an overview of the limitations of existing approaches, followed by some open issues which have not received much attention from researchers. Finally, Section 5 concludes the paper.

## 2. Flying Robots Serving People and a WSN

This section discusses some common issues when FRs serve non-collaboration objects. In particular, some basic models used in different applications are presented first, and then typical issues from the optimization point of view are discussed.

### 2.1. Coverage Model

Coverage is usually the first issue the system designers need to consider. In different applications, there are different meanings of coverage. Here, the coverage concept is divided into three cases: camera coverage, charging coverage and communication coverage. In particular, communication coverage is further classified into two categories. This classification is due to the different system assumptions. Specifically, when interference is not considered in the system, a simple coverage similar to the camera coverage is adopted; while when interference is accounted, a more precise mechanism is necessary to characterize the coverage.

#### 2.1.1. Camera Coverage

**Figure 2:**
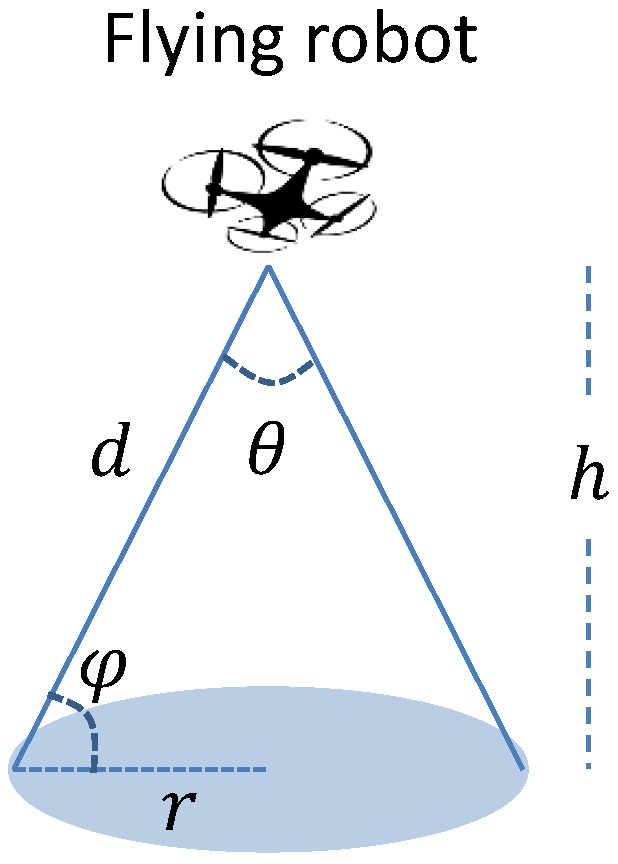
Disk coverage model.

In the application of target tracking, FRs are usually equipped with cameras facing the ground [13,22,23]. In general, the cameras have a fixed angle of vision θ, see Figure 2. Then, when a FR is at altitude *h*, the corresponding coverage area is a circular area with the radius:

(1) $$r_{max}=h\tan\left(\frac{\theta }{2}\right).$$

If a target is within the range of $r_{max}$ to the projection of a FR, this target is covered by the FR [7,22,24,25]. It is clear that the coverage radius relates to the altitude of the FR, and increasing the altitude enlarges the coverage area. An implied assumption is that the target has the Line-of-Sight (LoS) with the FR. If there is any obstacle between the two parts, the former cannot be observed by the latter, although the above condition holds.

#### 2.1.2. Charging Coverage

Another coverage model is based on the similar idea to the camera coverage, and it is suitable for applications of wireless charging sensor nodes by FRs [26]. Unlike many other power-harvesting methods, such as solar and vibration, radio frequency power harvesting (RF-power harvesting) can recharge multiple devices simultaneously, and it is not significantly dependent on the environment. However, the received power and the efficiency of the harvesting module of RF-power harvesting are both highly dependent on the distance between the charger and node. In [26], the energy-harvesting efficiency by a node depends on two terms: the received power and the efficiency of the harvesting antenna. Both of them depend on the distance between the charger and the node. For the former, a prorogation model proposed in [27] is adopted, where the received power decreases with increasing distance. For the latter, the efficiency values provided by the manufacturer Powercast [28] are used. Consider a scenario where a FR can charge a ground sensor node for a limited period. Taking into account the sensor nodes’ energy consumption model, and setting the objective as fully replenishing the nodes’ battery, one can obtain the maximum distance $d_{max}$ between a flying charger and a sensor node [26]. For a given FR at some position and under the above setting, a node can work without time limit if it is within $d_{max}$ of a flying charger.

#### 2.1.3. Communication Coverage I

In the applications of providing wireless communication service to cellular users, the coverage of a user by a FR can be determined by the signal pathloss (PL). Beyond the LoS assumption, references [29,30] consider that the links between FRs and ground users can have two cases: LoS and Non-LoS (NLoS). The authors of [30] propose a model for the probability of having LoS link between the two parts ($P_{LoS}$), which depends on the elevation angle, and some environmental parameters:

(2) $$P_{LoS}=\frac{1}{1+a\exp(-b(\varphi -a))},$$

where φ is the elevation angle (see Figure 2), and *a* and *b* are environment dependent parameters. As pointed out by [30], *a* and *b* depend on environmental parameters including the ratio of built-up land area to the total land area, the mean number of buildings per unit area and a scale parameter that describes the buildings’ heights distribution according to Rayleigh probability density function. The probability of NLoS link is $P_{NLoS}=1-P_{LoS}$. Furthermore, the pathloss is modelled with two parts: free space pathloss and excessive pathloss $\eta_{\xi }$, where ξ∈{LoS,NLoS}. Free space pathloss depends on the distance between the FR and the ground user, while excessive pathloss depends on the type of link between the two parts. Thus, the average pathloss from the FR to the ground user is the sum of the LoS pathloss and NLoS pathloss [30]:

(3) $$PL=P_{LoS}PL_{LoS}+P_{NLoS}PL_{NLoS},$$

where $PL_{\xi }=20\log\left(\frac{4\pi fd}{c}\right)+\eta_{\xi }$, *d* is the Euclidean distance between the FR and the ground user, *f* is the carrier frequency and *c* is the speed of light. Furthermore, by setting a maximum allowed pathloss, one can compute the largest coverage radius for a given altitude. Furthermore, the optimal altitude, which corresponds to the global largest coverage radius, can also be obtained [30].

#### 2.1.4. Communication Coverage II

**Figure 3:**
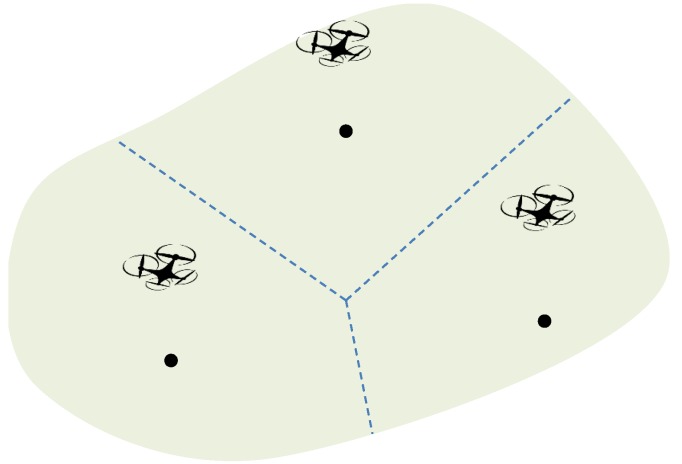
Coverage areas based on Vonoroi cells.

One implied assumption of the communication coverage model I is that only one FR is used to serve ground users, or multiple FRs use different wireless channels. Regarding this, a more practical communication coverage model should take interference into account. In the communication coverage model I, the coverage of a user or the association of it with an FR is determined by the maximum allowed pathloss or signal-to-noise ratio (SNR). When interference is accounted, the coverage or association should be determined by signal-to-interference-and-noise ratio (SINR). Following the average pathloss model [30], it can be seen that the received signal from the nearest FR is always the largest. Thus, by assuming that the association of a user and a FR is determined by SINR, a user is always associated with the nearest FR [31]. Obviously, the area of interest can be separated into several Voronoi cells given the positions of FRs, see Figure 3.

To briefly sum up, although the first three coverage models may be suitable to varying scenarios, the common feature of them is that the coverage area is a disk, which is independent of the position of the FR. Conversly, the coverage area of the last model depends on the relative positions of nearby FRs.

### 2.2. Connectivity

Many applications involving FRs require that they form a connected network. For example, in the application of data collection from wireless nodes using FRs, the connectivity of FRs with the central data sink guarantees that sensory data can be delivered to the data sink quickly, which makes it possible that end users take necessary actions timely. The case is the same in data dissemination. Another application is using FRs to provide communication service to ground cellular users. The connectivity of FRs with ground SBSs ensures that every FR has a wireless backhaul link so that any request from users can be transmitted to the core networks instantly and the response can also be returned to the user shortly. The connectivity requirement significantly influences the deployment of FRs, especially in disaster areas. In such areas, all or most of the existing SBSs may be destroyed by the disaster, thus, the FRs need to construct a new communication system and connect themselves to the remote working SBSs.

To this end, one simple model to characterize the connectivity requirement has been proposed in [32]. Consider a communication system consisting of *n* FRs and *m* SBSs, and the FRs are working at the same altitude. Let $P_{1},P_{2},\ldots ,P_{n}$ be the coordinates of FRs on the horizontal plane and $Q_{1},Q_{2},\ldots ,Q_{m}$ be the fixed locations of SBSs. The connectivity of such a communication system can be described by a communication graph G [32]. In the given communication graph G, there are n+m vertices and any robot vertex should be connected to an SBS vertex. The connectivity of two robot vertices and one robot vertex and one SBS vertex can be described by:

(4) $$D\left(P_{i},P_{j}\right)\leq R_{1},$$

if robot vertices *i* and *j* are connected by an edge in G; and

(5) $$D\left(P_{i},Q_{j}\right)\leq R_{2},$$

if robot vertex *i* and SBS vertex *j* are connected by an edge in G, where D(·,·) denotes the 2D distance between two vertices, and $R_{1}$ and $R_{2}$ are given constants. Based on the idea of [32], 3D connectivity can be obtained easily by introducing the altitude dimension [13,33].

Besides the connectivity discussed here, which focuses on the FRs and SBSs, another concept relating to connectivity is that FRs can work as relays to link disconnected networks. Such a concept is not covered in this survey and interested readers are referred to [34] and the references therein.

### 2.3. Energy Consumption

It is clear that many current commercial FRs are powered by onboard batteries, which means that their working time is limited. Some publications focus on flight control to improve energy efficiency and increase working time [35,36]. One model describing energy consumption of a FR is as follows [7,26]:

(6) $$E=\left(\beta +\alpha h\right)t+P_{max}\left(h/s\right)$$

where *E* is the energy consumption during the period of *t*, β is the minimum power needed to hover just over the ground, α is a motor speed multiplier, *s* is the lifting speed, and $P_{max}\left(h/s\right)$ is the energy spent for lifting the robot to altitude *h* with the speed *s*. A similar model has been discussed in [23]. Superior to [7,26], the model for recharging FRs is also presented in [23], but the idea is similar. Another model characterizing the energy consumption for flying is given in [37] and such a model simply assumes that the energy consumption for flying is proportional to the flying distance. Readers are referred to [36], where a more complex energy consumption model is discussed.

### 2.4. Coverage Cptimization

With the models of coverage, connectivity, and energy consumption, in this subsection, some typical optimization problems are discussed.

#### 2.4.1. Maximizing the Number of Covered Targets

For the case with a given number of FRs, if they are not sufficient to cover all the targets, one common objective is to maximize the number of covered targets. In general, the problems of maximizing the number of covered targets can be classified into two categories based on the input information about the users: exact location and general distribution.

Assuming the availability of targets’ locations, a few publications provide some formulations for the deployment of a single FR [38,39]. Specifically, an energy-efficient deployment approach for covering the maximum number of targets is presented in [38]. It first sets the vertical position of the FR at the altitude providing the maximal coverage and then optimizes the horizontal position to maximize the number of covered targets while using minimum transmitting power. Instead of decoupling the deployment, reference [39] formulates a mixed-integer non-linear problems involving both the horizontal coordinates and the altitude as variables, to maximize the number of served targets. Under the assumption of knowing targets’ locations, the case of multiple FRs has also been studied [40]. FRs are used to serve the maximum number of targets, subject to that each robot has a capacity of service. The authors propose a K-mean clustering base algorithm. The reference [41] considers FR deployment problem with the objective of maximizing target coverage and minimizing the moving distance of FRs when they need to change positions. A heuristic algorithm and a linear programming-based method are proposed.

For the case of multiple FRs, another type of input information is the general distribution of targets [6,32,42]. It is worth mentioning that such a distribution differs from the long-term traffic behavior, which is usually used for the deployment of SBSs. The distribution considered here is for occasional events, such as sports games and concerts. The concept of target density is usually used to describe the distribution. Let ρ(p) denote the target density of the location p∈S, where *S* is the area having targets to be covered by FRs. Following the communication coverage model I, the total coverage area of the set of FRs can be represented by $C(P_{1},\ldots ,P_{n})\in S$. Therefore, the maximization of the number of covered targets can be formulated as follows:

(7) $$\max_{P_{1},\ldots ,P_{n}}\int_{p\in C(P_{1},\ldots ,P_{n})}\rho \left(p\right)dp.$$

In particular, reference [32] considers two objectives: the maximum covered number of targets and the energy consumption by FRs for data transmission, together with the connectivity of FRs with SBSs. Reference [6] follows the framework of [32] to address another optimization problem. The first goal is the maximization of the number of covered targets. The authors also consider the recharging of FRs as well as interference management. Since this reference is based on the assumption of a street graph, the result is somewhat restrictive in practice.

#### 2.4.2. Minimizing Robot-User Distance

Another commonly considered optimization problem is to minimize the average robot-target distance [31]. To formulate this problem, the communication coverage model II is often used. Let $D_{min}\left(p\right)$ denote the distance from point p∈S to the nearest FR, which can be computed by [31]:

(8) $$D_{min}\left(p\right)=\min_{i=1,\ldots ,n}D\left(p,P_{i}\right).$$

Different from the communication coverage model I used in [32], the association of a target with an FR in [31] is based on distance. Then, the objective to minimize the average robot-target distance can be formulated as follows:

(9) $$\min_{P_{1},\ldots ,P_{n}}\int_{p\in S}\rho \left(p\right)D_{min}^{2}\left(p\right)dp.$$

where ρ(p) is the target density at position p∈S as mentioned above.

There are also some other publications based on target locations. Assuming the availability of target coordinates, the center of these targets can be computed by taking the mean value of the coordinates [43]. Then, a FR equipped with a tracking controller can track this center. Obviously, the center of targets achieves the minimum average distance between targets and the robot. Reference [44] aims at boosting network capacity using FRs. The authors propose a game-theory-based navigation algorithm. This algorithm is decentralized, but the computing load may be high since it uses exhaustive search to find a potential moving direction. Both these methods are based on the assumption of knowing the coordinates of all the targets. However, how to obtain such information is not answered in them. Almost all the location-based approaches have not addressed such an issue either. In practice, measuring the coordinates is quite difficult or at least costly if some specific protocols are used. One solution is to estimate the locations of targets. During the communication process with targets, the FRs can measure the received signal strength [45,46], from which the robots can estimate the locations of targets in a dynamic manner using the robust extended Kalman filter [47]. Based on the estimated locations, a decentralized reactive navigation algorithm is presented in [48]. Different from [43], the FRs in [48] move towards the weighted centers of mass. In other words, each user is assigned a dynamic weight and such weight depends on the distance between itself and the nearest robot. This is practical in real applications since a user which is closer to a robot will generally receive better quality of service than a user further from the robot. Although [48] does not assume knowing the user locations, which is superior to [43] and many other location-based approaches, the performance of [48] depends on the accuracy of the location estimation. Another reactive approach is based on virtual forces [49]. The authors assume that the FRs are able to recognize users by onboard sensors when they fly. They consider four types of virtual forces: hotspots attractive force, user attractive force, nearby robot repulsive force, and obstacle repulsive force. One weakness of [49] is that the locations of hotspots should always be detected before deploying the FRs.

#### 2.4.3. Minimizing the Number of Flying Robots

The above approaches are about the deployment of a given number of FRs. A different objective is to figure out the minimum number of required FRs to provide a high quality of communication service or target coverage, which relates to the system investment. In [25], the authors deploy the FRs at the same altitude to cover a set of targets, given their locations. Based on the disk coverage model, they formulate a Geometric Disk Cover problem with the objective of using the minimum number of robots to cover all the targets. To address the problem, a centralized heuristic algorithm is proposed. Beyond the 2D situation considered in [25], the authors of [50] consider the case of deploying FRs in 3D space with the same objective and a PSO-based heuristic algorithm is proposed. Reference [51] studies the similar problem and an elitist non-dominated sorting genetic algorithm is used to find the optimal positions for FRs from a given set of candidates.

Besides the application of supporting wireless communication using FRs, the minimization of FR number has also been considered in other applications such as wireless charging sensor nodes [26] and target tracking [7,24,37]. Publications [7,24] study the continuous camera coverage problem. The objectives are to minimize the number of FRs and energy consumption. Reference [7] formulates a mixed-integer non-linear optimization model and presents a mixed-integer programming-based heuristic algorithm. Paper [24] considers the similar case as [7] and the authors further propose a localized heuristic algorithm beyond the centralized one in [7]. It is worth mentioning that the advantages of [7,24] include the consideration of the energy limitation of FRs. Paper [37] integrates the recharging requirements into the continuous coverage problem and examines the minimum number of FRs for covering multiple subareas. The authors partition the coverage graph into cycles that start from the charging depot and the number of FRs required depends on the charging time, the traveling time, and the number of subareas to be covered by the cycle.

#### 2.4.4. Minimizing Energy Consumption

Since many FRs are powered by the onboard batteries, minimizing energy consumption improves operating lifetime. Also, when the serving object of FRs is a WSN, the energy consumption of sensor nodes is often considered as a key point to ensure a long enough network lifetime.

Reference [52] considers the energy-efficient deployment of a FR to provide wireless communication to targets. The received power at a target from a FR is modelled as a function of the horizontal distance between the target and the FR, and the altitude of the FR [52]. Then, paper [52] decouples the optimal position in the horizontal and vertical dimensions. The horizontal position is taken as the center of the smallest circle covering the given set of targets, and then the vertical position is obtained such that the minimum transmitting power is used to cover that circle. Publication [23] focuses on the application of area monitoring. With a given set of FRs and a charging depot on the ground, the authors consider the problem of how to schedule the FRs to execute either monitoring task or recharging task, such that the network lifetime is maximized. They follow the camera coverage model and the energy consumption model therein is similar to Equation (6). Another recent publication using a single FR is to prolong the network lifetime of a WSN [53]. Instead of minimizing the energy consumption of the FR, the main points here are scheduling the wake-up time of sensor nodes and planning the trajectory of the FR, such that the maximum energy consumption of sensor nodes is minimized.

#### 2.4.5. Other Optimization Problems

Wireless charging sensor nodes in WSNs is a promising way to improve the lifetime of WSNs. Recent survey [54] reviews the related approaches using ground mobile robots as wireless chargers. In this subsection, some typical approaches using FRs for wireless charging sensor nodes are reviewed.

Reference [55] presents some fundamental results related to charging sensor nodes using FRs. A simple scenario where a single FR is used, and it can charge a single sensor at any time. With the objective of maximizing network lifetime, the authors aim at selecting appropriate nodes to recharge as well as the best sink node selection for data collection. Beyond this, the case where a FR can recharge multiple sensor nodes has also been considered. Reference [56] considers the scenario of using a set of FRs to collect data from a set of sensors and recharge them simultaneously. An optimization problem with the objective of maximizing data collection utility is proposed. A one-side-matching algorithm and a greedy algorithm are proposed to address the problem. Publication [57] moves beyond [56] by considering the varying energy consumption rates of sensors. A dynamic charging strategy is presented for a single FR with the objective of minimizing overall packet loss rate. Paper [58] focuses on the joint scheduling of charging routes and sensor association for multiple FRs. The authors formulate a bounded route association problem and an approximation algorithm is proposed. The authors of [26] formulate a set cover problem to figure out the 3D positions of FRs, with the objective of minimizing the FR number such that all the sensor nodes can operate without time limitation.

### 2.5. Summary

In this section, some typical approaches for FRs in various applications are discussed. To make a brief summary, a thorough comparison of these approaches by several common metrics is made, including the problem dimension, the robot number, the robot deployment manner, and the type of input information that the system uses for positioning the FRs. In particular, the dimension of a problem can be 1D, 2D, and 3D, which depends on applications. The number of FRs can be single or multiple. The robot deployment manner can be divided into proactive and reactive. Generally, proactive deployment refers to the off-line approaches, which compute the positions for the FRs in advance; by contrast, the reactive deployment refers to the online approaches, which can dynamically calculate robots’ positions. For the type of information the system uses, two groups can be considered: location and density. The discussed approaches are summarized in Table 1. Table 1 presents the features of the discussed approaches under the aforementioned metrics and briefly summarizes the objectives of the corresponding optimization problems. Such a table may assist designers in picking the most useful approach according to the system features.

## 3. Flying Robots Collaborating with Ground Objects

Section 2 discussed the approaches in one aspect of IoFR, i.e., the applications involving objects which passively accept service from FRs. This section concentrates on the other aspect of IoFR, where objects can collaborate with FRs for some complex tasks. Two types of systems are discussed: a flying-ground robotic system, and the system where FRs collaborate with a WSN.

### 3.1. Flying-Ground Robotic System

Many applications are enabled by the collaboration of flying and ground robots, such as goods delivery [59], mapping [60], surveillance [61], and the exploration of unknown environments [62]. Exploring unknown environments using autonomous robots has been regarded as a fundamental problem in robotics applications such as search and rescue and 3D modelling. A basic requirement of these applications is to scan unknown space or detect free space in the shortest time. In the literature, both ground robots [63,64,65] and FRs [66,67,68,69] have been employed for such a task. These two entities have their own features. Ground robots can carry heavy and long-range laser scanners which may be impossible for FRs with weight constraints. Although FRs have limited flying time, they can have better mobility and agility, since they can fly above obstacles and view areas that are inaccessible to ground robots [70].

One well-studied application regarding exploring unknown environments is search and rescue after disasters. It is clear that some areas may be well explored in normal periods, but they may not be the same after disasters. Thus, to search-and-rescue victims, such areas should be seen as unknown environments and the search-and-rescue team should be able to operate in these environments. There are several key issues involved in this application: searching targets, planning path, and reaching a target.

#### 3.1.1. Target Search

After disasters, the first task is to search for victims needing rescue. Considering that the areas may still have potential risks, autonomous robots have been widely used in this operation. Ground robots were first used to search targets in unknown environments [71] where the tasks of the ground robot included simultaneous localization and mapping. Furthermore, the ground robot team explored and performed target-searching operation [72,73,74]. Compared to ground robots, FRs are agile and fast. The growing recognition of the potential of using FRs for search-and-rescue operations is supported by an increasing number of approaches in image recognition for victim detection [75,76,77]. A key factor to be considered in image recognition is the quality of sensory data [78]: the probability of false negative, i.e., the probability of missing a victim, should remain low; and the probability of false positive, i.e., the probability of recognizing a non-victim object, should also be low. Furthermore, when a team of FRs is used, data sharing influences the efficiency of searching. Data fusion is necessary to generate a complete picture of the environment; and the connectivity of FRs with ground rescue team is also necessary to be considered as a trade-off with the wideness of the search area. Obviously, always requiring connectivity will narrow the searching range of FRs.

#### 3.1.2. Path Planning and Target Reaching

Once the target is found, the next task is to plan a valid path towards the target for the rescue team. Traditional graph searching algorithms such as Dijkstra’s algorithm [79], $A^{*}$ [80], and many of their extensions, which were originally designed for global optimal path planning, cannot be used, due to their heavy computation load. Since the environments involved in rescue tasks may be dynamic, e.g., because of the collapse of buildings, the rescue team should be able to avoid dynamic obstacles reactively. In other words, efficient online algorithms are required. There are some available approaches for reactive navigation of ground robots, such as [81,82,83,84,85,86]. Readers are referred to survey [87,88] for more details.

#### 3.1.3. Integrated Systems

Some research groups have also focused on integrated flying-ground robotic systems, combining two entities to improve overall system performance. By making use of their advantages and shortening their disadvantages, the capabilities that every single robot is unable to achieve can be obtained [62,89,90]. In the flying-ground robotic system of [62], the FRs are regarded as the backup entity and they are used only in the case of areas invisible to ground robots. The authors of [89,90] present a system consisting of a ground robot and a FR for search-and-rescue missions in unknown environments.

In the system of [89], the FR first surveys the environment in a lawn-mower fashion and searches the target. Then, it sends the position of the target back to the ground robot. After that, the FR returns to the starting point and signals the ground robot to move towards the target following a collision-free trajectory generated by a LiDAR. While the ground robot is heading towards the target by following the trajectory, the FR follows the former autonomously and keeps it within the camera view, so that the remote human operator can detect a hazardous situation in time and take control of the system in case of an emergency, which cannot be handled by flying-ground robotic systems autonomously. Thus, such a system is an autonomous one except for the participation of humans in an emergency.

In the system of [90], the remote operator participates in the target search task. Once the target is found, the FR engages in autonomous vision-guided flight to a series of waypoints that are actively chosen. For each waypoint, 3D reconstruction and ground robot path are computed, and the next waypoint is chosen in a way that minimizes the estimated response time (including aerial exploration and ground path traversal time). This system uses an “on-the-spot training” approach to classify terrains [91] and REMODE for 3D reconstruction [92]. Then, the traversing time to move to the next waypoint can be estimated, based on the information of terrain type and the elevation gradient (both of which influence the actual speed of the ground robot).

As well as the aforementioned application, other applications such as field inspection and parcel delivery using a flying-ground robotic system have also attracted much attention. The key point in these applications is using one or more FRs to visit a given set of points. Considering the constrained onboard battery capacity, the flight time of a FR is limited. One idea is to construct some ground charging stations where a FR can recharge or swap the battery [93]. The deployment of the ground charging stations influences the coverage of the FRs. Under the assumption of having a set of ground charging stations, the authors of [94] focus on path planning of a FR, with the goal of visiting a given set of targets successfully. The other idea is to use ground robots as mobile charging platforms [60,95,96]. In particular, the authors of [60] consider using a battery-constrained FR and a battery-unlimited ground robot for large-scale mapping applications. The FR can recharge its battery on the ground robot. While the ground robot can only move on the road network, the FR can traverse the areas off the road network. The authors provide a strategy for the cooperation of the FR and ground robot such that they can finish the mapping mission under the constraint so that the FR never runs out of battery. The authors of [95] consider a similar scenario as [60] and they provide an integer program for the problem. Different from [60,95], in the system of [96], the FR can travel with the ground robot together and recharge its battery during the movement. Clearly, this strategy reduces the time to finish the mission.

### 3.2. Flying Robots Collaborating with a WSN

Different from the approaches discussed in Section 2, where FRs are used as mobile sinks (mobile chargers) to collect sensory data (deliver energy) from (to) sensor nodes, this subsection discusses another application where WSNs provide service to FRs, such as localization and navigation.

#### 3.2.1. Localization of Flying Robots

A typical example is that FRs are used to monitor key plants in the indoor factory environment, such as nuclear power stations, which is safer, more efficient, and more economical than human patrol. There are several challenges to be addressed about the navigation of FRs. In indoor environments, GPS may not work, which makes localizing FRs a challenge, as well as navigation. Although a camera can to be equipped on the FRs, it will lead to a heavy computing load because of image processing, which may be impossible for micro FRs with low computing performance [97]. To this end, WSNs can be used to localize FRs in indoor environments using the extended Kalman filter and time difference of arrival (TDOA) measurements of radio signal [98].

#### 3.2.2. Navigation of Flying Robots

In WSNs, replacing a failed sensor node is a typical operation. To achieve this by FRs, the capability to navigate a FR towards a sensor node is required. One approach is based on received signal strength indication (RSSI) [99,100]. Specifically, in reference [99], the target sensor node periodically sends out beacons, and the FR can measure RSSI to determine the moving direction. Under the similar setting, reference [100] presents a reduced particle filtering method, which is well suited for devices with limited computational power and energy resources.

The authors of [101] propose another system for navigating FRs. Here, the destination of a FR may not be a specific sensor node, but some other places in the industrial environment. The system consists of: (1) a set of sensor nodes, equipped with 3D range-finder sensors, to detect the dynamic obstacles such as vehicles and walking people, (2) micro FRs, equipped with tracking controller only, and (3) a central controller. The micro FRs should measure their positions and directions and send such information to the central controller. The sensor nodes should also send their measuring data to the central controller. Based on the two types of information, a safe path is generated for each FR by the central controller. Then, the FRs, equipped with a simple tracking controller, track those paths to their destinations.

A similar publication to [101] is [102], where a team of FRs perform surveillance without possessing sensors with automated target recognition capability, and thus rely on communicating with unattended ground sensors placed on roads to detect and image potential intruders. The authors focus on the path-planning problem with the objective of maximizing the likelihood of a FR and an intruder being at the same location.

### 3.3. Summary

To briefly sum up, this section has discussed some basic tasks when FRs collaborate with ground robots and WSNs. The former focuses on the application of search and rescue. The operations of target searching, path planning, and navigation are discussed. The latter mainly considers the localization and navigation of FRs using a WSN. The discussed approaches are summarized in Table 2.

## 4. Discussions and Future Research Directions

This section makes some discussions about the aforementioned approaches. At the same time, the discussions can raise some promising future research directions.

### 4.1. Connectivity Consideration

As mentioned above, when multiple robots are used, each robot needs to have a valid wireless backhaul link at any time, to guarantee the delay of response, or form a connected backbone to transmit the collected sensory data to SBSs. References [31,32,33] have addressed this issue by requiring each robot to be connected to an SBS either directly or via another for relay, but the issues related to the data flow have not been covered in them. Under this model, the system will work as long as the connectivity is set up. However, the data rates at the one-hop robots are different from those with two hops when serving users [13]. Therefore, such connectivity is not guaranteed to provide satisfactory service. One possible solution is inspired by the uneven clustering problem in WSNs [103,104], i.e., setting different serving numbers for the FRs with different hops, i.e., an unequal association between users and robots. In this way, the one-hop robots can serve a smaller number of users than the robots with two or more hops. Then, the one-hop robots can have more resources to relay the requests from the robots which are connected to it.

Another drawback of [31,32] is that they all fix the topology at first and then find optimal positions for FRs satisfying this topology. It is clear that the topology of robots can also be optimized to achieve no worse performance of coverage. References [13,33] consider finding a subset of positions for the FRs from a set of candidates. It is easy to understand that this method can generate different topologies of connected graphs, which is superior to [31,32].

### 4.2. Optimal Deployment in 3D Space

Among the discussed approaches, some are based on grids [7,24]. Although discretization simplifies the problem, the performance of the solution depends highly on the resolution of grids. The higher resolution makes the solution closer to the optimal one, but it increases the computing load, while the lower resolution makes the searching computational efficient, but the solution may be far from the optimal. Furthermore, there are some approaches based on the formulation of mixed-integer programming, such as [13,22,33,105]. One common feature of them is the assumption that the possible positions of FRs are given by a set of candidates. These candidates can be regarded as a special set of grids.

From this discussion, it is believed that some efficiently computational algorithm should be developed for a case with continuous 3D deployment space.

### 4.3. Reactive Deployment of Flying Robots

It can be seen from Table 1 that most available approaches for wireless communication support, target monitoring, etc., are proactive; while only a few are reactive. Obviously, the reactive approaches are more suitable for dynamic situations. Thus, much more research effort should be made on the development of reactive deployment methods. Furthermore, many of the existing publications assume the availability of targets’ exact locations. It is clear that they are difficult to collect in practice, which impedes the applications of these methods. Therefore, a promising research direction is to develop location-free deployment algorithms.

### 4.4. Navigation with Collision Avoidance

In the application of search and rescue, navigation with collision avoidance is important for both ground and FRs. Approaches such as [81,83,84,106] and some others such as [107,108,109,110] have proposed various path planning and reactive navigation algorithms. Although some of them are designed for FRs, such as [107,108,109], a fixed altitude is assumed; while few papers studied the much more difficult case of collision-free 3D navigation [111,112,113,114,115]. With this regard, there is a necessity to extend the available 2D methods to the 3D scenario. Furthermore, 3D risk-aware navigation is more preferred to avoid no-flight areas, such as populated areas and those with chemical plants [107,108,109]. Risk-aware navigation especially suits urban environments, where a relatively larger number of obstacles and no-flight areas exist. Another important direction for future research is to obtain some 3D versions of barrier and sweep coverage problems [116] for monitoring and surveillance applications.

### 4.5. Charging Flying Robots

Though many papers have presented strategies for FRs in various applications, only a few of them have considered the battery recharging issue. Typically, small FRs have fuel constraints, which prevent them from being used for long-term or large-scale missions.

Similar to the idea of using a ground robot as a mobile charging platform [60,95,96], an interesting research direction is to involve public transportation vehicles such as buses into the system. The FRs can stay on the top of these vehicles, travel together with them, and charge themselves simultaneously. Then, they can take off at some positions and head to their targets unreachable by these vehicles. This idea may simplify the flying-ground robotic system, since the task is to plan paths for FRs only. However, to make such a system operate efficiently, the timetables and the uncertainties in the timetables, e.g., due to the traffic congestion in peak hours, should be accounted for in the planning stage.

## 5. Conclusions

In this paper, the applications of the Internet of Flying Robots (IoFR) were investigated. Several typical problems across the applications such as wireless communication support, target tracking, data collection and dissemination from or to WSNs, collaborating with ground robots, etc., were discussed. Specifically, the coverage issue, the connectivity issue, the energy limitation constraint, target searching, path planning, collision avoidance and FR navigation were the main focus of this survey. The available publications for these issues were reviewed and comparisons were made to illustrate the features of them, through which shortcomings were pointed out. Some potential methods to address unsolved problems were indicated and some promising research directions were discussed.

This paper mainly focused on some high-level topics of IoFR. Some other aspects which are related to IoFR, such as communication techniques between robots and the low-level control techniques, are not covered.

## Figures and Tables

**Table 1:** Summary of typical approaches for FRs serving humans and WSNs.

Approach	FR Number	Dimension	Proactive or Reactive	Density, Location, or Distance-Based	Remark
[30]	Single	1D	Proactive	Location	Altitude optimization for coverage area
[52]	Single	3D	Proactive	Location	Minimizing transmitting power
[38]	Single	3D	Proactive	Location	Minimizing transmitting power
[39]	Single	3D	Proactive	Location	Maximizing covered user number
[43]	Single	2D	Reactive	Location	Tracking the center of users
[53]	Single	2D	Proactive	Location	Prolong WSN network lifetime
[25]	Multiple	2D	Proactive	Location	Minimizing robot number
[32]	Multiple	2D	Proactive	Density	Maximizing covered user number
[6]	Multiple	2D	Proactive	Density	Interference management
[37]	Multiple	2D	Proactive	Location	Recharge sensor nodes in cycle
[50]	Multiple	3D	Proactive	Location	Minimizing FR number
[51]	Multiple	3D	Proactive	Location	Minimizing FR number, maximizing data rate
[42]	Multiple	2D	Proactive	Density	Neural-based cost function
[31]	Multiple	2D	Proactive	Density	Decentralized robot-user distance minimization; connectivity
[40]	Multiple	2D	Proactive	Location	K-means clustering
[33]	Multiple	3D	Proactive	Location	Minimizing FR number; connectivity
[7]	Multiple	3D	Reactive	Location	Minimizing FR number; energy constrained
[44]	Multiple	2D	Reactive	Location	Exhaustive search moving direction
[48]	Multiple	2D	Reactive	Distance	Move towards weighted centers
[49]	Multiple	2D	Reactive	Location	Navigation based on virtual force
[55]	Single	2D	Proactive	Location	Selection charging node and sink node
[56]	Multiple	2D	Proactive	Location	Maximization of data collection utility
[57]	Single	2D	Proactive	Location	Varying energy consumption rates
[58]	Multiple	2D	Proactive	Location	Charging routes and sensor association
[26]	Multiple	3D	Proactive	Location	Minimizing FR number

**Table 2:** Summary of typical approaches for FRs collaborating with ground robots and WSNs.

Approach	Task	Collaboration Type
[71]	Target searching using a ground robot	FRs-ground robots
[72,73,74]	Target searching using a ground robot team	FRs-ground robots
[75,76,77]	Target searching using FRs	FRs-FRs
[81,82,83,84,85,86]	Reactive navigation for ground robots	FRs-ground robots
[89,90]	Flying-ground robotic search-and-rescue team	FRs-ground robots
[60,95,96]	Field inspection and parcel delivery with charging stations	FRs-ground robots
[98]	Localization of FRs by a WSN	FRs-WSN
[99,100]	FR navigation based on RSSI in WSNs	FRs-WSN
[101,102]	FR navigation by sensory information	FRs-WSN

## Abbreviations

The following abbreviations are used in this manuscript:

IoFR	Internet of Flying Robots
FR	Flying Robot
WSN	Wireless Sensor Network
SBS	Stationary Base Station
LoS	Line-of-Sight
SNR	Signal-to-Noise Ratio
SINR	Signal-to-Interference-and-Noise Ratio
RSSI	received signal strength indication
